# Left intraventricular pressure gradient in hypertrophic cardiomyopathy patients receiving implantable cardioverter-defibrillators for primary prevention

**DOI:** 10.1186/s12872-021-01910-0

**Published:** 2021-02-19

**Authors:** Kyoichiro Yazaki, Atsushi Suzuki, Tsuyoshi Shiga, Yuichiro Minami, Kotaro Arai, Kyomi Ashihara, Morio Shoda, Nobuhisa Hagiwara

**Affiliations:** 1 Department of Cardiology, Tokyo Women’s Medical University, Tokyo, Japan; 2 Department of Clinical Pharmacology and Therapeutics, The Jikei University School of Medicine, 3-25-8 Nishi-shinbashi, Minato-ku, Tokyo, 105-8461 Japan; 3 Clinical Research Division for Heart Rhythm Management, Tokyo Women’s Medical University, Tokyo, Japan

**Keywords:** Hypertrophic cardiomyopathy, Implantable cardioverter-defibrillator, Sudden cardiac death, Risk, Ventricular arrhythmia

## Abstract

**Background:**

Conventional risk factors for sudden cardiac death (SCD) justify primary prevention through implantable cardioverter-defibrillator (ICD) implantation in hypertrophic cardiomyopathy (HCM) patients. However, the positive predictive values for these conventional SCD risk factors are low. Left ventricular outflow tract obstruction (LVOTO) and midventricular obstruction (MVO) are potential risk modifiers for SCD. The aims of this study were to evaluate whether an elevated intraventricular pressure gradient (IVPG), including LVOTO or MVO, is a potential risk modifier for SCD and ventricular arrhythmias requiring ICD interventions in addition to the conventional risk factors among HCM patients receiving ICDs for primary prevention.

**Methods:**

We retrospectively studied 60 HCM patients who received ICDs for primary prevention. An elevated IVPG was defined as a peak instantaneous gradient ≥ 30 mmHg at rest, as detected by continuous-wave Doppler echocardiography. The main outcome was a composite of SCD and appropriate ICD interventions, which were defined as an antitachycardia pacing or shock therapy for ventricular tachycardia or fibrillation. The Cox proportional hazards model was used to assess the relationships between risk factors and the occurrence of SCD and appropriate ICD interventions.

**Results:**

Thirty patients met the criteria of elevated IVPG (50%). During the median follow-up period of 66 months, 2 patients experienced SCD, and 10 patients received appropriate ICD interventions. Kaplan–Meier curves showed that the incidence of the main outcome was higher in patients with an IVPG ≥ 30 mmHg than in those without an IVPG ≥ 30 mmHg (log-rank *P* = 0.03). There were no differences in the main outcome between patients with LVOTO and patients with MVO. The combination of nonsustained ventricular tachycardia (NSVT) and IVPG ≥ 30 mmHg was found to significantly increase the risk of the main outcome (HR 6.31, 95% CI 1.36–29.25, *P* = 0.02). Five patients experienced ICD implant-related complications.

**Conclusions:**

Our findings showed that a baseline IVPG ≥ 30 mmHg was associated with an increased risk of experiencing SCD or appropriate ICD interventions among HCM patients who received ICDs for primary prevention. Combined with NSVT, which is a conventional risk factor, a baseline IVPG ≥ 30 mmHg may be a potential modifier of SCD risk in HCM patients.

## Background

Hypertrophic cardiomyopathy (HCM) is a common and genetically heterogeneous form of cardiomyopathy characterized by unexplained left ventricular (LV) hypertrophy [1–3]. In general, many patients with HCM have a normal life expectancy, and they do not require major therapeutic interventions [1]. However, sudden cardiac death (SCD) occurs at an annual rate of 1% among adults with HCM [4]. According to the current guidelines, five conventional risk factors for SCD justify primary prevention through implantable cardioverter-defibrillator (ICD) implantation [2, 3]. However, the positive predictive values for these conventional SCD risk factors are low (10–20%) [2, 5]. The 2011 American College of Cardiology Foundation (ACCF)/American Heart Association (AHA) guidelines suggest that the presence of some established risk factors alone is sufficient to warrant ICD implantation, while others recommend ICD implantation when these factors exist in conjunction with other SCD risk factors or modifiers [2].

LV outflow tract obstruction (LVOTO) is known to be a potential risk modifier for SCD [6–8]. However, the annual rate of SCD among HCM patients with LVOTO is reported to be 1.0–1.5% and the positive predictive value of LVOTO for SCD is quite low (7–9%) [6–8]. Moreover, the annual rate of SCD in HCM patients with LVOTO and no other risk is reported to be quite low (0.37%) [6]. To determine the appropriate method of selecting high-risk patients for ICDs, further evaluations of HCM patients with LVOTO and the presence of additional risk factors are needed. As shown in our previous study, LV obstruction at the midventricular level, known as midventricular obstruction (MVO), may also be a potential predictor for SCD and lethal arrhythmias [9]. We also reported that 15 HCM patients with MVO and the presence of conventional major risk factors who received ICDs for primary or secondary prevention for SCD experienced appropriate ICD interventions at an annual rate of 6.2% [10].

Therefore, we aimed to evaluate whether an elevated intraventricular pressure gradient (IVPG) (defined as ≥ 30 mmHg at rest [6–10]), including LVOTO or MVO, is a potential risk modifier for SCD and ventricular arrhythmias requiring ICD interventions in addition to conventional risk factors among HCM patients receiving ICDs for primary prevention.

## Methods

### Patients and study design

**Fig. 1:**
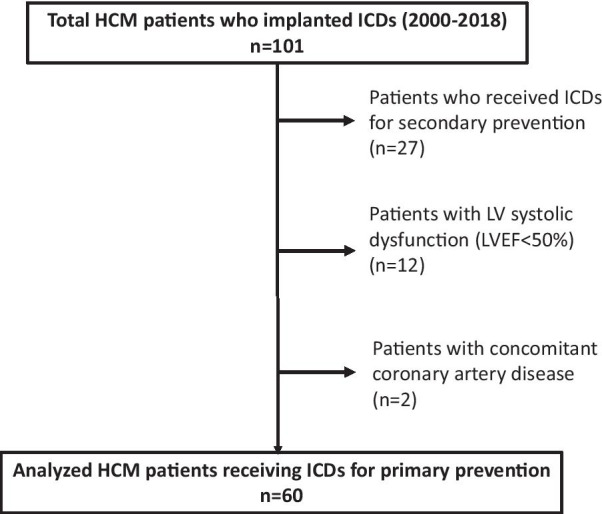
Flow diagram of the study subjects

We conducted a retrospective study with 101 consecutively treated HCM patients who underwent ICD implantation between 2000 and 2018 at Tokyo Women’s Medical University Hospital. HCM was diagnosed by the 2-dimensional echocardiographic identification of LV hypertrophy with a nondilated LV cavity in the absence of other cardiac or systemic causes of LV hypertrophy, such as Fabry disease or amyloidosis [1, 2]. We excluded patients who received ICDs for secondary prevention, including survivors of SCD and patients with sustained ventricular tachycardia (VT). We also excluded patients with LV systolic dysfunction (LVEF < 50%) and concomitant coronary artery disease. Ultimately, we included 60 HCM patients who received ICDs for primary prevention in this analysis (Fig. 1).

All patients were admitted to the Department of Cardiology at the Tokyo Women’s Medical University Hospital for an evaluation of HCM and the risk of SCD. Before ICD implantation, all patients underwent 12-lead electrocardiography (ECG), 24-h Holter ECG, treadmill stress testing, and echocardiography. In our institution, when cardiac catheterization is performed for the evaluation of HCM, a right ventricular (RV) endomyocardial biopsy is also performed for patients in whom it is required to differentiate secondary cardiomyopathy, unless there are contraindications. Thirty-one patients underwent an RV biopsy. ICDs were implanted for primary SCD prevention when patients presented with one or more of the following major conventional risk factors according to the following guidelines and consensus: unexplained syncope, family history of SCD, nonsustained ventricular tachycardia (NSVT), LV wall thickness ≥ 30 mm and abnormal blood pressure response to exercise [1, 2, 11]. An electrophysiological study was performed to make an independent judgment for "gray zone" patients who had only one or two major risk factors and were considering ICD implantation. Thirty-eight patients underwent an electrophysiological study. The protocol was approved by the Institutional Review Board of Tokyo Women’s Medical University.

### IVPG

All patients underwent echocardiographic studies before medical treatment and ICD implantation. Echocardiographic data were assessed by two independent echocardiographic specialists (KA1, KA2) with blinded to patient data. A significantly elevated IVPG was defined as an instantaneous peak of ≥ 30 mmHg detected by continuous-wave Doppler echocardiography while patients were at rest. In this study, IVPG provoked by the Valsalva maneuver, inhalation of amyl nitrate, exercise or with dobutamine infusion was not examined because our study included patients with HCM in the 1990–2000s and the Japanese guidelines first recommended the assessment of LV outflow tract gradient provoked by the Valsalva maneuver in 2018 [12]. LVOTO was defined as an estimated peak LV outflow tract gradient of ≥ 30 mmHg [8]. MVO was defined by the detection of hypertrophy of the mid-left ventricular walls and systolic obstruction of the mid-left ventricle with an estimated peak gradient of ≥ 30 mmHg [9, 13].

### Outcomes and follow-up

The main outcome was a composite of SCD and appropriate ICD interventions including antitachycardia pacing or shock therapy for VT and ventricular fibrillation (VF). SCD was defined as unexpected, endogenous death within 1 h after having been observed alive or unexpected, unwitnessed death during sleep that was unrelated to a specific cause of circulatory failure.

Follow-up visits were conducted every 3–6 months until December 2018 at our pacemaker or ICD clinic. The median follow-up period was 66 months, and the follow-up range was 40–100 months. The occurrence of VT or VF that required ICD therapies, including both shock and antitachycardia pacing, was determined from ICD interrogation reports stored on each device. Details of events and electrocardiograms were reviewed by 2 independent investigators (AS, TS). The VF detection zone (270–319 ms, number of intervals to detect 8/12) was programmed for all patients. The VT zone (375–429 ms, number of intervals to detect 16–24) was programmed for 51 patients, and the fast VT zone (331–351 ms) was programmed for 18 patients. ICD shock (defibrillation) occurred when ventricular arrhythmias were detected in the VF and high-speed VT zones. Antitachycardia pacing, including burst and/or autodecremental ramp pacing, was delivered when triggered by VT. A shock was delivered if the pacing failed to terminate VT. Antitachycardia pacing was programmed for 33 patients with NSVT or inducible sustained VT to avoid shock therapy. In our study, in patients for whom the VT zone was set, VT therapy (detection < 389 ms) was activated in 18 (30%) patients, and only the monitor zone (detection average 408 ms) was used in 33 (55%) patients. Patients were followed until death from any cause, loss to follow-up, or December 2018. Five patients were lost to follow-up. These patients had been followed for over 50 months after ICD implantation at our hospital but were subsequently referred to other hospitals in distant locations because of patient relocation. For this reason, these patients could not be followed until December 2018, and this study treated them as lost to follow-up.

### Statistical analysis

The summary data are presented as the number of patients, the mean ± standard deviation, or the median and interquartile range. Comparisons between groups were performed using Student’s *t*-test for normally distributed continuous variables (assessed by the Shapiro–Wilk test) and the Mann–Whitney *U* test for other variables. The categorical variables were subjected to chi-squared analysis. The cumulative proportions of the event-free rate were calculated using the Kaplan–Meier method. Differences in the event-free rates were compared using the log-rank test. Univariate analyses using the Cox proportional hazards model were performed to assess the relationships between the main outcome and IVPG ≥ 30 mmHg or the following risk factors/confounders: NSVT, unexplained syncope, family history of SCD, abnormal blood pressure response to exercise, LV wall thickness ≥ 30 mm, gadolinium enhancement on cardiac magnetic resonance imaging (CMR) and the high-risk criteria for the European Society of Cardiology (ESC) risk prediction model for sudden cardiac death in hypertrophic cardiomyopathy (HCM Risk-SCD score) [3]. The relative risk for an increase in one conventional risk factor for the main outcome was estimated by the Cox proportional hazards model. Univariate analysis using the Cox proportional hazards model was also performed to assess the association between each pair of risk factors and the main outcome. P values < 0.05 were considered significant. The data were analyzed using Statistical Package for the Social Sciences (SPSS) statistical software (version 22.0.0, SPSS, Inc., Chicago, Illinois).

## Results

### Patient characteristics

The baseline characteristics of the patients are shown in Table 1. The mean patient age was 55 ± 16 years. Six patients were < 30 years old at the time of ICD implantation. Thirty patients met the criteria for an elevated IVPG (50%). There was no significant difference in age, sex, conventional risk factors, HCM Risk-SCD score, CMR findings or echocardiographic parameters between patients with and without an IVPG of ≥ 30 mmHg. There was increased use of RV pacing and class I antiarrhythmic drugs in patients with an IVPG ≥ 30 mmHg. Fifty-three of 60 patients were administered beta-blockers as first-line drugs. The remainder of the patients were administered calcium channel blockers such as diltiazem and verapamil. Sixteen of our patients were also administered amiodarone (4 for atrial arrhythmia, 8 for ventricular arrhythmia and 4 for both arrhythmias).

**Table 1:** Characteristics of patients with and without left ventricular pressure gradient

	With IVPG of ≥ 30 mmHg (n = 30)	Without IVPG of ≥ 30 mmHg (n = 30)	*P*-value
Age (years)	56 ± 13	54 ± 18	0.55
Male	20 (67%)	22 (73%)	0.78
NYHA functional class I/II/III	14/13/3	17/13/0	0.19
NSVT	26 (87%)	22 (73%)	0.33
Unexplained syncope	11 (37%)	14 (47%)	0.60
Family history of sudden death	10 (33%)	9 (30%)	1.00
Abnormal BP response during exercise	6 (20%)	7 (23%)	1.00
Maximum LV wall thickness ≥ 30 mm	4 (13%)	3 (10%)	1.00
*Number of major conventional risk factors*			0.21
1	12 (40%)	10 (33%)	
2	9 (30%)	16 (53%)	
3	8 (27%)	4 (13%)	
4	1 (3%)	0	
*HCM risk-SCD score*			
5-year risk (%)	5.3 (3.1–8.1)	4.0 (3.0–5.9)	0.14
Category*			0.38
High risk	12 (40%)	6 (20%)	
Intermediate risk	7 (23%)	9 (30%)	
Low risk	11 (37%)	15 (50%)	
Cardiac magnetic resonance imaging	(n = 23)	(n = 17)	
LGE-positive	19 (83%)	15 (88%)	1.00
LV mass (g)	112 ± 44	107 ± 44	0.72
Electrophysiological study	(n = 15)	(n = 23)	
Inducible VT or VF	8 (53%)	6 (26%)	0.49
*Portion of LV obstruction*			
Outflow tract	18 (60%)		
Mid-ventricle	12 (40%)		
*Echocardiographic parameters*			
Left atrial dimension (mm)	37 ± 6	41 ± 9	0.10
LV end-diastolic dimension (mm)	43 ± 7	46 ± 7	0.19
LV end-systolic dimension (mm)	27 ± 7	29 ± 6	0.28
LV ejection fraction (%)	61 ± 11	62 ± 10	0.84
Maximum LV wall thickness (mm)	21 ± 7	19 ± 7	0.20
*Implantable cardioverter defibrillator*			
Subcutaneous type	0	1 (3%)	
Transvenous type	30 (100%)	29 (97%)	
Pacing mode			
AAI	0	1 (3%)	
VVI	3 (10%)	10 (35%)	
DDI	3 (10%)	1 (3%)	
DDD	24 (80%)	17 (59%)	
RV pacing > 95%	17 (57%)	2 (7%)	< 0.01
Tachycardia detection setting/therapy setting			
VF zone	30/30	30/30	
Fast VT zone	7/7	11/11	
VT zone	25/13	24/5	
*Medications*			
Beta-blockers	27 (90%)	26 (87%)	1.00
Calcium channel blockers	5 (17%)	4 (13%)	1.00
ACE inhibitors/ARBs	7 (23%)	19 (63%)	< 0.01
Class I antiarrhythmic drugs	11 (37%)	1 (3%)	< 0.01
Amiodarone	8 (27%)	8 (27%)	1.00

Values are presented as the numbers of patients (%), means ± SD, or median (interquartile range)

ACE, angiotensin-converting enzyme; ARB, angiotensin II receptor blocker; BP, blood pressure; HCM-Risk SCD, a risk prediction model for sudden cardiac death in hypertrophic cardiomyopathy; IVPG, intraventricular pressure gradient; LGE, gadolinium enhancement; LV, left ventricular; NSVT, nonsustained ventricular tachycardia; NYHA, New York Heart Association; RV, right ventricular; VT, ventricular tachycardia; VF, ventricular fibrillation

*High risk: 5-year risk ≥ 6%, Intermediate risk: 5-year risk ≥ 4% and < 6%, Low risk: 5-year risk < 4%

### SCD and ICD intervention

During the median follow-up period of 66 [range 40–100] months, 5 patients died, and their causes of death included SCD (2 patients), heart failure (1 patient), aortic dissection (1 patient) and pneumonia (1 patient). Regarding the main outcome, 2 patients (one with NSVT and unexplained syncope and another with NSVT alone as a risk factor) experienced SCD, and 10 patients received appropriate ICD interventions. Of the 2 patients who experienced SCD, one patient experienced appropriate ICD interventions associated with worsening heart failure symptoms before death. This patient had a baseline ESC score of 1.59 and reduced her IVPG from 30 to 0 mmHg using sotalol. The police contacted us to learn her disease profiles because she died at home, but we could not confirm the device data, so the rhythm at the time of death was unknown. Another patient was transferred to the emergency department due to cardiopulmonary arrest, where pulseless electrical activity was detected. This patient had a baseline ESC score of 6.22 and reduced his IVPG from 177 to 34 mmHg using disopyramide. Their LVEF was reduced to 40% before death.

Among the 10 patients who experienced appropriate ICD interventions, 45 episodes were terminated with antitachycardia pacing (3 episodes in the VF zone; 42 episodes in the VT zone), and 11 episodes were terminated with shock therapy during the follow-up period. The composite rate of SCD and appropriate ICD interventions was 3.1%/year. There were no differences in SCD or appropriate ICD interventions between patients with LVOTO and patients with MVO (5/18 vs 4/12, respectively, *P* = 0.745).

During the follow-up period, NSVT within the VT zone was detected in 13 patients, and SCD or appropriate ICD interventions were observed in more patients in whom NSVT was detected than in those in whom NSVT was not detected (5/13 vs 4/38, *P* = 0.02). Six patients progressed to the overt dysfunction phase (defined by an LVEF < 50%), and SCD or appropriate ICD interventions were observed in more patients who progressed to the overt dysfunction phase than in those who did not (4/6 vs 7/54, *P* = 0.01).

Among the patients in our study, 19 used RV pacing with a shortened AV delay to decrease the IVPG, especially the LV outflow tract gradient. In approximately 80% (n = 15) of the patients, the IVPG was successfully reduced to < 30 mmHg using RV pacing. However, a reduced IVPG (< 30 mmHg) after RV pacing did not decrease the incidence of SCD or appropriate ICD interventions (HR 1.35, 95% CI 0.58–3.28).

Additionally, 10 patients experienced inappropriate ICD interventions for the following reasons: atrial fibrillation (n = 7), sinus tachycardia (n = 1), and noise sense (n = 1). Five patients experienced ICD implant-related complications, including infection requiring the removal of the defibrillator (n = 1), cardiac tamponade (n = 1), lead displacement (n = 2), and pocket bleeding (n = 1).

### IVPG and conventional risk factors

**Fig. 2:**
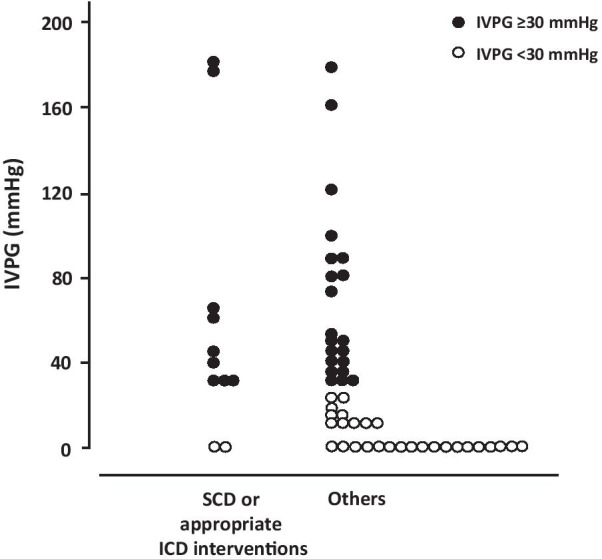
Absolute values of the left intraventricular pressure gradient (IVPG) among hypertrophic cardiomyopathy patients who met the criteria of an elevated IVPG (≥ 30 mmHg) with and without sudden cardiac death (SCD) or appropriate implantable cardioverter-defibrillator (ICD) interventions

**Fig. 3:**
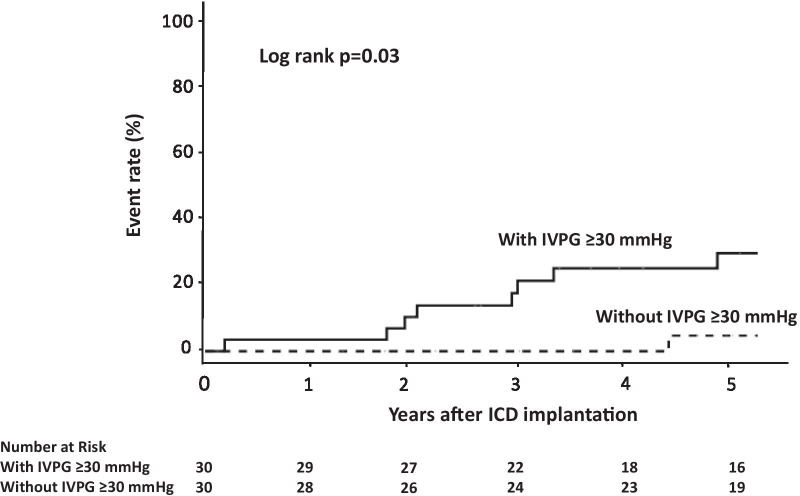
Kaplan–Meier analysis of the main composite outcome of sudden cardiac death and appropriate implantable cardioverter-defibrillator interventions among hypertrophic cardiomyopathy patients presenting with and without an elevated left intraventricular pressure gradient (IVPG)

The median IVPG and 5-year SCD risk of HCM Risk-SCD score in 11 patients who experienced SCD or appropriate ICD interventions were 40 [25–64] mmHg and 6.1 [5.1–6.5] %, respectively. The distribution of IVPG between patients with and without SCD or appropriate ICD interventions is shown in Fig. 2. A baseline IVPG ≥ 30 mmHg was observed in 9 of 11 patients with SCD or appropriate ICD interventions and 21 of 49 patients without SCD or appropriate IC1D interventions. The Kaplan–Meier curves for the main outcome showed that a higher incidence was observed among patients with an IVPG ≥ 30 mmHg than among patients without an IVPG ≥ 30 mmHg (Fig. 3). The univariate Cox analysis of risk factors related to the main outcome is shown in Additional file 1: Table S1.

According to our results, a single conventional risk factor did not contribute to the composite main outcome of SCD and appropriate ICD interventions. The number of risk factors was also not associated with the risk of the main outcome (relative risk per increase of one factor 0.97 [0.45–2.13]). In this study, 38 patients (63%) underwent an EP study. However, inducible sustained VT/VF did not predict the main outcome (HR 0.45, 95% CI 0.08–2.71). Among the patients in our study, 6 of 18 patients who met the high-risk criteria (estimated as a 5-year SCD risk of > 6%) according to the ESC HCM Risk-SCD score and for whom ICD implantation was recommended as the primary prevention of SCD [3], received ICD interventions but did not show a significantly higher incidence of the main outcome (Additional file 1: Table S1).

**Fig. 4:**
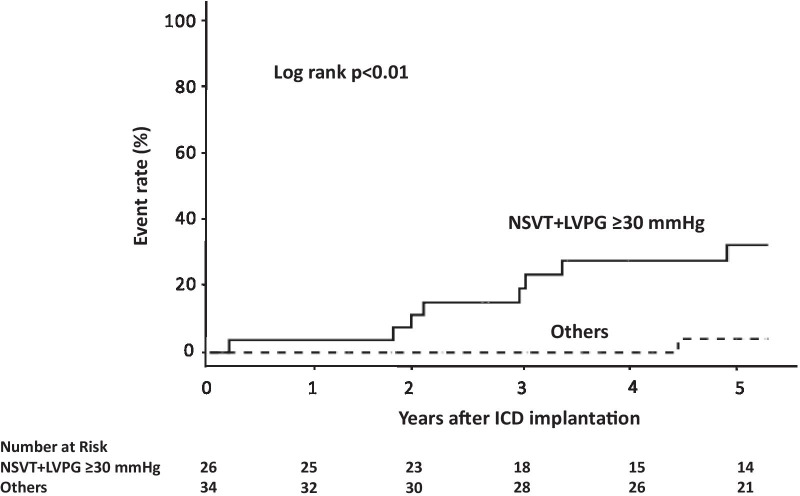
Kaplan–Meier analysis of the main composite outcome of sudden cardiac death and appropriate implantable cardioverter-defibrillator interventions among hypertrophic cardiomyopathy patients presenting with and without nonsustained ventricular tachycardia (NSVT) and an elevated left intraventricular pressure gradient (IVPG)

Furthermore, because 80% of the patients in our study who received ICDs presented with NSVT, which is an established risk factor, the risk ratio for the combination of NSVT and an IVPG ≥ 30 mmHg was statistically significant for the main outcome (Table 2). A statistically significant interaction effect was not observed when other pairs of factors were considered (Additional file 2: Table S2). The Kaplan–Meier curves for the main outcome are shown in Fig. 4. A significantly higher incidence was observed among patients with NSVT and an IVPG ≥ 30 mmHg than among other patients.

**Table 2:** Hazard ratio for association between risk factors combined with NSVT and the main composite outcome of SCD and appropriate ICD interventions

	Events/patients		HR (95% CI)	*P*-value
	With risk	Without risk		
NSVT + unexplained syncope	4/19	7/41	1.12 (0.33–3.84)	0.86
NSVT + family history of SCD	4/10	7/50	3.40 (0.99–11.66)	0.05
NSVT + LV wall thickness ≥ 30 mm	1/6	10/54	0.74 (0.10–5.82)	0.78
NSVT + abnormal BP response	1/8	8/40	0.58 (0.07–4.61)	0.60
NSVT + LGE on CMR	5/26	1/16	3.31 (0.38–38.56)	0.28
NSVT + IVPG ≥ 30 mmHg	9/26	2/34	6.31 (1.36–29.25)	0.02

BP, blood pressure; CI, confidence interval; CMR, cardiac magnetic resonance imaging; HR, hazard ratio; IVPG, intraventricular pressure gradient; LGE, gadolinium enhancement; LV, left ventricular; NSVT, nonsustained ventricular tachycardia; SCD, sudden cardiac death

## Discussion

Our study revealed several important findings, as described below. (1) Half of the patients with HCM who received ICDs for the primary prevention of SCD met the criteria of an elevated IVPG, which was defined as an instantaneous peak ≥ 30 mmHg. (2) Patients with a baseline IVPG ≥ 30 mmHg exhibited an increased risk of experiencing SCD or appropriate ICD interventions, which was the main composite outcome. (3) There were no differences in SCD or appropriate ICD interventions between patients with LVOTO and patients with MVO. (4) The combination of NSVT and an IVPG ≥ 30 mmHg was associated with a statistically significant risk of experiencing SCD or appropriate ICD interventions.

In this study, the composite rate of SCD and appropriate ICD interventions was 3.1%/year in patients with HCM who received ICDs for primary prevention. According to a meta-analysis, 85% of patients with HCM who received ICDs for primary prevention experienced appropriate ICD interventions at a rate of 3.3%/year [14]. It should be noted that not all of the appropriate ICD therapies were lifesaving because some of the ICD therapies might be delivered for ventricular arrhythmias that have self-terminated. During the past 2 decades, ICD programming has been adapted according to the recommendations of trials and guidelines [15, 16], and the detection duration of shipment programming is also different according to the manufacturer. It was a limitation of this study that there was variability in the setting in the VT zone and therapy. Furthermore, a meta-analysis also reported that inappropriate ICD interventions and ICD-related complications occurred at nontrivial annual rates of 4.8% and 3.4%/year, respectively, in HCM patients who received ICDs [14]. For each patient, the potential for primary prevention of SCD should be considered with more careful risk stratification.

### IVPG and conventional risk factors

In our study, an IVPG ≥ 30 mmHg was associated with an increased risk of experiencing SCD or appropriate ICD interventions, which was the main composite outcome. According to some studies, LVOTO (a resting gradient of ≥ 30 mmHg) might be associated with the risk of SCD among HCM patients [6, 7]. However, other studies have failed to reveal an association between resting LVOTO and SCD [17, 18]. Because LVOTO varies on a diurnal and day-to-day basis and because exercise-induced augmentation of the gradient cannot be differentiated from resting measurements, the usefulness of LVOTO (a resting gradient ≥ 30 mmHg) is limited as an independent risk factor for SCD. Similar findings have been reported for MVO in HCM patients. Because data on MVO in HCM patients have been reported in studies with small sample sizes only [9, 10, 19], researchers have not clearly confirmed that this structural abnormality is a significant risk factor for SCD. Although these data are limited, this abnormality should be considered during the development of SCD risk assessment strategies [2, 3]. In our study, there were no differences in SCD or appropriate ICD interventions between patients with LVOTO and patients with MVO. Therefore, there seems to be no definite clinical significance in classifying patients with an elevated IVPG based on LVOTO and MVO. While it is often difficult to distinguish between types of intra-LV cavity obstruction in patients with HCM in clinical practice, an elevated IVPG is not difficult to detect using the Doppler method.

On the other hand, dual-chamber pacing is often used to reduce IVPG in patients with LVOTO to improve their symptoms. The recent guidelines state that dual-chamber pacing improves symptoms and quality of life in patients with LVOTO through a reduction in LV outflow tract gradients but that there are no data regarding whether a reduction in IVPG caused by dual-chamber pacing decreases the risk of SCD in patients with HCM [2, 3]. In our study, which included a small sample size, a reduced IVPG caused by RV pacing did not show a significant benefit in terms of preventing SCD or ICD interventions. To confirm this postulation, additional well-designed research studies with adequate sample sizes are necessary.

Five established conventional risk factors are associated with the need for primary prevention [1, 2, 20, 21]. The negative predictive values for each risk factor for the need for primary prevention are generally high, but the positive predictive values for each risk factor are quite low [2]. In our study, we were unable to identify a single risk factor that is associated with SCD or appropriate ICD interventions among patients with HCM who received ICDs. The risk of SCD has been reported to increase with the number of risk factors [22, 23]. However, a significant relationship between the number of risk factors and the rate of appropriate ICD interventions has not been shown in other reports [20]. In our study, the number of risk factors was not clearly associated with an increased risk of experiencing SCD or appropriate ICD interventions. Recently the HCM Risk-SCD has been used for the risk stratification of patients with HCM for primary prevention of SCD. The LVOT gradient, a component of elevated IVPG, is included in this calculation. In this study, patients with a high HCM Risk-SCD score failed to show significant results despite a relatively large hazard ratio value because the study included only a small number of high-risk patients. As validated in large samples [24], this score is considered a useful risk predictor for general HCM patients.

In 2 patients who experienced SCD, one patient had a high HCM Risk-SCD score, and the other patient had a low score. One patient with an IVPG of 0 mmHg was assumed to have died due to worsening heart failure and another whose IVPG was 34 mmHg died due to pulseless electrical activity. The causes of SCD in HCM are not only VT and VF, but also asystole, pulseless electric activity and high-grade atrioventricular block [25]. Although it is uncommon, progressive LV dysfunction occur in some HCM patients, and patients with an overt dysfunction phase (defined by an LVEF < 50%) show an incidence of SCD exceeding 10% per year [26]. Among the 6 patients who progressed to overt dysfunction in our study, 5 patients had a baseline IVPG ≥ 30 mmHg, 2 patients experienced SCD and the other 2 patients received appropriate ICD interventions. Elevated IVPG did not directly contribute to the development of VT or VF as a cause of SCD in these 2 patients, but a baseline IVPG ≥ 30 mmHg in patients with HCM might be a factor that contributes to disease progression, resulting in worsening heart failure or myocardial ischemia.

### Combination of NSVT and IVPG ≥ 30 mmHg

Among the conventional risk factors, NSVT is commonly exhibited by patients with HCM during ambulatory ECG monitoring [22, 27–31]. In our study, 80% of the patients who received ICDs had NSVT as an established risk factor. However, considering NSVT to be a risk factor for SCD remains controversial. The 2011 ACCF/AHA guidelines recommend that patients aged > 30 years with HCM who present with NSVT and other SCD risk factors or modifiers should be considered for prophylactic ICD implantation [2]. We evaluated the effects of risk factors combined with the presence of NSVT and found that the combination of NSVT and an IVPG ≥ 30 mmHg was a better potential risk marker for SCD or appropriate ICD interventions than either factor alone. HCM with elevated IVPG is likely to cause increased LV wall stress and myocardial ischemia upon exertion. Moreover, additional NSVT may act as a trigger in sustained VT/VF. Therefore, these electrical and structural abnormalities might contribute to the development of sustained VT or VF in patients with HCM who present with NSVT and elevated IVPG.

In our institution, ≥ 2 conventional risk factors or 1 conventional risk factor plus ≥ 1 risk modifier (intraventricular obstruction, atrial fibrillation, left atrial dimension, or induced sustained VT/VF) has been the primary indication of ICD for SCD prevention. Our previous reports showed that the incidence of NSVT in HCM patients was 30–40% [32, 33]. Therefore, NSVT is most commonly found as a conventional risk factor in HCM patients in screening examinations for SCD. As a result of ICD implantation for primary prevention based on this policy, NSVT was most common, followed by IVPG ≥ 30 mmHg, which was more common than other conventional risk factors. In addition, the combination of NSVT and IVPG ≥ 30 mmHg highly predicted subsequent arrhythmia events in HCM patients who received ICDs. An ICD may be useful for selected HCM patients with NSVT and IVPG ≥ 30 mmHg. To evaluate the predictive role of the combination of IVPG ≥ 30 mmHg with other conventional risk factors, a large-scale cohort is required.

### Study limitations

This study has some limitations. A retrospective, observational design was used in this study, and this study was performed at a single center. Consecutively treated patients were enrolled in the study to minimize selection bias. However, 5 patients were lost to follow-up because they moved, which could have biased the results of the study. Our study population was limited to select high-risk HCM patients who received ICDs for the primary prevention of SCD. Therefore, we have not yet clearly determined whether our findings are generalizable to HCM patients who do not receive ICDs. We also did not include patients who received ICDs as a secondary preventative measure from the viewpoint of risk stratification. To confirm our hypothesis, a further study with broad cohort of HCM patients is needed.

Data regarding the patients’ clinical conditions at the time of the ICD intervention were not available. Additionally, treatment bias existed. Furthermore, the number of subjects was relatively small, and therefore, a subgroup analysis was not feasible.

## Conclusions

Our findings showed that a baseline IVPG ≥ 30 mmHg was associated with an increased risk of experiencing SCD or appropriate ICD interventions among HCM patients who received ICDs for primary prevention. The combination of NSVT and an IVPG ≥ 30 mmHg was a good potential risk predictor. A baseline IVPG ≥ 30 mmHg may be a potential risk modifier for SCD in HCM patients.

## Supplementary Information

**Additional file 1: Table S1.** Univariate analysis for the main composite outcome of SCD and appropriate ICD interventions.

**Additional file 2: Table S2.** *P*-values for each pair of established conventional risk factors, LGE on CMR and IVPG ≥ 30 mmHg. This table shows the *P*-values obtained from the univariate analysis using the Cox proportional hazards model, which was performed to assess the association between each pair of risk factors and the main composite outcome of SCD and appropriate ICD interventions.

## Data Availability

The data that support the findings of this study are available from the corresponding author upon reasonable request.

## Abbreviations

ACCF: American College of Cardiology Foundation
AHA: American Heart Association
CMR: Cardiac magnetic resonance imaging
ECG: Electrocardiogram/electrocardiography
ESC: European Society of Cardiology
HCM: Hypertrophic cardiomyopathy
HCM Risk-SCD score: Risk prediction model for sudden cardiac death in hypertrophic cardiomyopathy
ICD: Implantable cardioverter-defibrillator
IVPG: Intraventricular pressure gradient
LV: Left ventricular
LVOTO: Left ventricular outflow obstruction
MVO: Midventricular obstruction
NSVT: Nonsustained ventricular tachycardia
RV: Right ventricular
SCD: Sudden cardiac death
VT: Ventricular tachycardia
VF: Ventricular fibrillation
