# Supplementary material for: Left intraventricular pressure gradient in hypertrophic cardiomyopathy patients receiving implantable cardioverter-defibrillators for primary prevention
Source: BMC Cardiovasc Disord. 2021 Feb 19;21:106. doi: 10.1186/s12872-021-01910-0 (PMC7893864; doi:10.1186/s12872-021-01910-0)
Supplement: Supplementary file 1 — Additional file 1: Table S1. Univariate analysis for the main composite outcome of SCD and appropriate ICD interventions. [file 12872_2021_1910_MOESM1_ESM.docx]

Additional file 1: Table S1. Univariate analysis for the main composite outcome of SCD and appropriate ICD interventions.

Events / patients

With risk Without risk HR (95% CI) P-value

IVPG ≥30 mmHg 9/30 2/30 4.82 (1.04-22.36) 0.04

NSVT 11/48 0/12 29.50 (0.06-1368) 0.28

Unexplained syncope 4/25 7/35 0.69 (0.20-2.37) 0.55

Family history of SCD 4/19 7/41 1.27 (0.37-4.34) 0.70

Abnormal BP response 1/13 8/35 0.28 (0.04-2.25) 0.23

LV wall thickness ≥30 mm 1/7 10/53 0.59 (0.08-4.79) 0.64

LGE on CMR 5/34 1/6 1.21 (0.19-23.41) 0.86

HCM Risk-SCD Score: High risk 6/18 5/42 2.42 (0.73-7.96) 0.15

BP, blood pressure; CI, confidence interval; CMR, cardiac magnetic resonance imaging; HCM-Risk SCD, a risk prediction model for Sudden Cardiac Death on Hypertrophic Cardiomyopathy; HR, hazard ratio; IVPG, intraventricular pressure gradient; LGE, gadolinium enhancement; LV, left ventricular; NSVT, nonsustained ventricular tachycardia; SCD, sudden cardiac death
