# Supplementary material for: Left intraventricular pressure gradient in hypertrophic cardiomyopathy patients receiving implantable cardioverter-defibrillators for primary prevention
Source: BMC Cardiovasc Disord. 2021 Feb 19;21:106. doi: 10.1186/s12872-021-01910-0 (PMC7893864; doi:10.1186/s12872-021-01910-0)
Supplement: Supplementary file 2 — Additional file 2: Table S2. P-values for each pair of established conventional risk factors, LGE on CMR and IVPG ≥ 30 mmHg. This table shows the P-values obtained from the univariate analysis using the Cox proportional hazards model, which was performed to assess the association between each pair of risk factors and the main composite outcome of SCD and appropriate ICD interventions. [file 12872_2021_1910_MOESM2_ESM.docx]

Additional file 2: Table S2.

P-values for each pairs of established conventional risk factors, LGE on CMR and IVPG ≥30 mmHg.

|  | NSVT | Syncope | Family history of SCD | LV wall thickness >30 mm | ABPR | LGE on CMR | IVPG  ≥30 mmHg |
| --- | --- | --- | --- | --- | --- | --- | --- |
| NSVT |  | 0.86 | 0.05 | 0.78 | 0.60 | 0.28 | 0.02 |
| Syncope |  |  | 0.34 | 0.62 | 0.61 | 0.46 | 0.17 |
| Family history of SCD |  |  |  | 0.52 | 0.77 | 0.61 | 0.23 |
| LV wall thickness >30 mm |  |  |  |  | － | 0.54 | 0.91 |
| ABPR |  |  |  |  |  | 0.61 | 0.68 |
| LGE on CMR |  |  |  |  |  |  | 0.29 |
| IVPG  ≥30 mmHg |  |  |  |  |  |  |  |

ABPR, abnormal blood pressure response; CMR, cardiac magnetic resonance imaging; IVPG, intraventricular pressure gradient; LGE, gadolinium enhancement; LV, left ventricular; NSVT, nonsustained ventricular tachycardia; SCD, sudden cardiac death

This table shows the P-values obtained ​​from the univariate analysis using the Cox proportional hazards model, which was performed to assess the association between each pair of risk factors with the main composite outcome of SCD and appropriate ICD interventions.
